# Pseudophakic retinal detachment in young-aged patients

**DOI:** 10.1371/journal.pone.0184187

**Published:** 2017-08-31

**Authors:** Thomas Laube, Claudia Brockmann, Nils Lehmann, Norbert Bornfeld

**Affiliations:** 1 Centre for Ophthalmology Düsseldorf, Düsseldorf, Germany; 2 Institute of Medical Informatics, Biometry and Epidemiology, University of Duisburg-Essen, Essen, Germany; 3 Department of Ophthalmology, University Hospital, University of Duisburg-Essen, Essen, Germany; Medizinische Universitat Graz, AUSTRIA

## Abstract

**Objective:**

To investigate the incidence and risk factors for retinal detachment (RD) after cataract surgery or refractive lens exchange (RLE) in patients aged below 61 years.

**Methods:**

Retrospective medical chart review of 7,886 patients (13,925 eyes) who underwent cataract surgery or RLE. Patients aged below 61 years were selected. Age, gender, axial length, follow-up times, and the occurrence of RD were recorded. Additional characteristics documented for RD cases were: history of RD, preexisting retinal findings, laser capsulotomy, status of macula at RD, date and details of RD.

**Results:**

From a total of 421 patients (677 eyes) aged below 61 years 24 cases of RD were identified, resulting in an overall cumulative incidence per eyes of 3.55%. The mean follow-up time was 45 ± 32.8 months. Ninety-two % of all RDs occurred within 3.6 years from surgery. Axial length had a significant effect on the risk of RD after cataract/ RLE surgery (HR = 1.42, P = 0.0001, 95% CI 1.19–1.69). The highest incidence of RD occurred in the subgroup of 25 to 28.9 mm axial length (10.2%). With an increase in age of ten years, the hazard of postoperative RD was not significantly increased by a factor of 1.50 (P = 0.286, 95% CI 0.71–3.15). The highest incidence of RD occurred in patients aged 50–54 years (5.39%). Compared to females, males had an almost twofold not significant risk of postoperative RD (HR = 1.96, P = 0.123, 95% CI 0.83–4.63). None of the RD cases had a history of RD.

**Conclusions:**

Axial length is a significant risk factor for pseudophakic RD. The need for cataract surgery or RLE should be carefully considered in patients with axial lengths between 25 and 29 mm, aged 50–54 years, in males, and in case of preexisting retinal findings.

## Introduction

Retinal detachment (RD) is a sight-threatening complication of cataract surgery. Multiple risk factors for RD have been described. These include predisposing patient factors (myopia [1–6], young age [1, 6–8], male gender [8–10], history of eye trauma [10]), surgical technique [4], surgical complications like vitreous loss, posterior capsule rupture [11, 12], and postoperative factors (neodymium:YAG laser posterior capsulotomy [1, 13, 14]).

While the incidence for RD in the general population has been estimated to range between 0.005% and 0.0179% [15–19], the incidence of RD after cataract extraction varies considerably according to the surgical technique, the length of follow-up, and study group characteristics [3, 20]. Improvement of cataract surgery techniques from intracapsular cataract extraction (ICCE) to extracapsular cataract extraction, and currently to phacoemulsification has reduced the incidence of RD after cataract surgery. RD incidences have been reported to vary between 1% and 8.1% after ICCE and follow-up times between 1 week and 22 years [20]. According to other authors incidences of RD after ICCE ranged between 0.4–3.6% [19]. Incidences of RD after extracapsular cataract extraction have been estimated to range between 0–7.5% after a follow-up time between 4 months and 18 years [20], or to vary between 0.55–1.65% [19]. Recent studies of cataract surgery using phacoemulsification found overall RD rates of 0.7% over 21 years [21], and an estimated RD risk of 0.99% at 4 years after surgery [10].

Though cataract surgery techniques continue to improve with reduced incision sizes and introduction of femtosecond laser assisted cataract surgery, increasing numbers of cataract operations generally in particular in younger patients may contribute to an increase of pseudophakic RDs in coming years. As younger age is one of the known risk factors for RD after cataract surgery, this study focuses on a young-aged group of patients undergoing lens extraction performed by one surgeon (T. L.), evaluating the incidence of RD in relation to axial length (AL), gender, and age subgroups.

## Patients and methods

**Fig 1:**
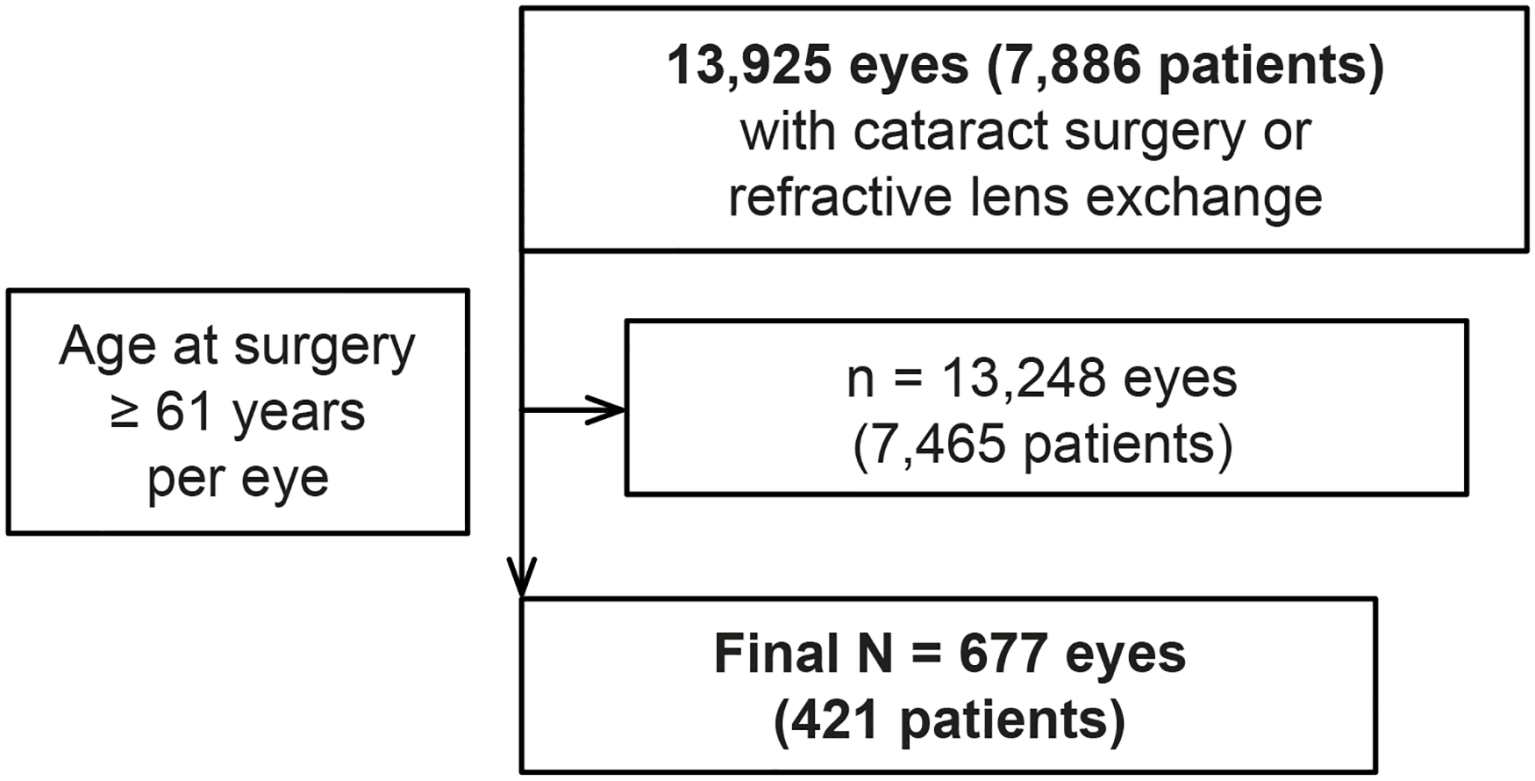
Flow chart showing selection of the study group based on the exclusion criterion “age at surgery ≥ 61 years per eye”.

The electronic patient records of 13,925 eyes from 7,886 patients undergoing cataract surgery or refractive lens exchange (RLE) between April 2004 and December 2014 at the Centre for Ophthalmology Düsseldorf were reviewed and selected for eyes of patients younger than 61 years at surgery (Fig 1). All surgeries were performed by the first author. The retrospective study was performed in accordance with the Declaration of Helsinki and approved by the Ethics Committee of the Medical Faculty of the University of Duisburg-Essen (approval number: 17-7403-BO). Written consent for data processing was obtained from all patients.

Demographic characteristics from the selected patients as well as AL, follow-up times, and the occurrence of RD were reviewed and recorded. Follow-up covered the period from cataract surgery (or RLE) to last examination, contact, or death of the patient. A minimum follow-up period of five years was intended for all cases with corresponding surgery date. Patients with follow-up less than five years were contacted to obtain information about the occurrence of RD. This was not possible in cases of death or unavailability of the patient or missing data on the treating ophthalmologist. In cases of RD, surgery reports and clinical data were requested from the corresponding clinic and the treating ophthalmologist.

The standard surgical technique was temporal clear corneal small incision cataract surgery with phacoemulsification and implantation of a posterior chamber hydrophobic acrylic intraocular lens. The self-sealing incision was performed with a width of 3.2 mm in 2004 and was subsequently reduced to 2.75 and 2.2 mm.

For cases diagnosed with RD the following characteristics were documented: history of RD, cryocoagulation, argon laser coagulation or posterior neodymium:YAG capsulotomy, occurrence of preexisting retinal scars or retinal degeneration, surgical complications, implanted IOL type, status of fellow eye, status of macula at RD, interval from surgery to RD and from capsulotomy to RD, best corrected visual acuity (BCVA) after cataract surgery/ RLE, findings of RD, details of RD repair surgery, and BCVA after repair of RD. Mean visual acuity was calculated using logMAR units.

Cox-regression models accounting for within patient variation using a random effect model as implemented in SAS (Cary, N.C.) statistical software version 9.4 were used to estimate the hazard ratio (HR, 95% confidence interval (CI)) for the time to RD associated with possible risk factors, as AL, age, and sex. RD event rates related to categorical variables were estimated using the Kaplan-Meier method and statistically evaluated using the log-rank test. Differences between groups with and without RD were evaluated using the two-tailed t-test or Mann-Whitney rank sum test. Chi-square statistics or the Fisher exact test was used to test the association between two categorical variables. Descriptive statistical analysis was conducted using the SPSS statistical software package, version 24. *P* values less than 0.05 were considered statistically significant.

## Results

**Fig 2:**
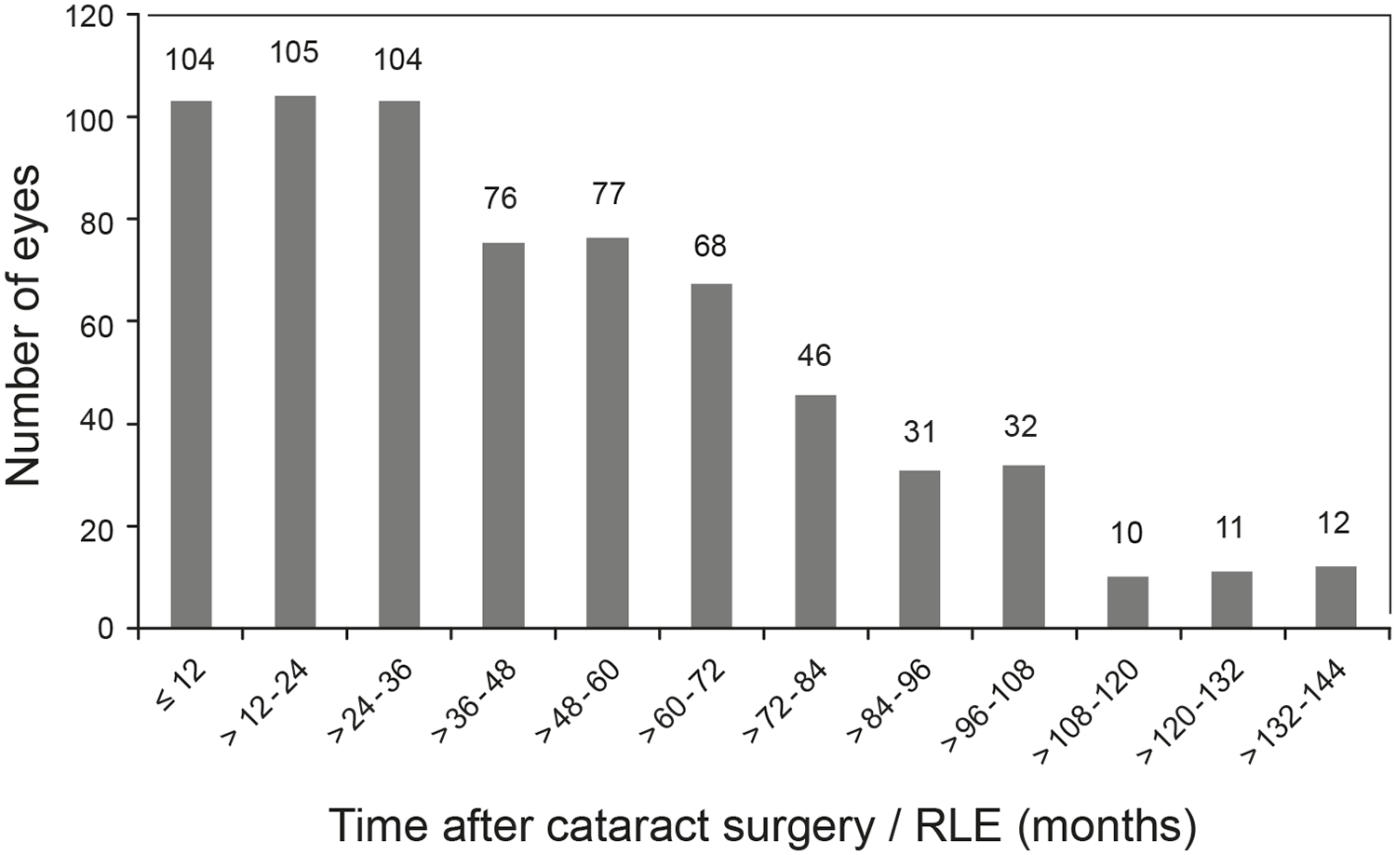
Follow-up times of all cases by number of eyes.

From the total number of 13,925 eyes (7,886 patients) that had cataract surgery or RLE, 677 eyes from 421 patients younger than 61 years were selected. The selected age group included 328 eyes from males and 349 eyes from females. The age at surgery ranged from 13.9 to 60.7 years with a mean age of 51.12 ± 6.78 years (each eye was counted separately). The AL of the eyeball was available for 629 eyes and ranged from 20.20 to 33.97 mm with a mean length of 24.36 ± 2.02 mm. Cataract surgery was performed in 585 eyes and 92 eyes underwent RLE. The mean follow-up time regarding eyes was 45 ± 32.80 months (range 1 day– 140 months; Fig 2). The total follow-up time was 2.537 years per eyes.

**Fig 3:**
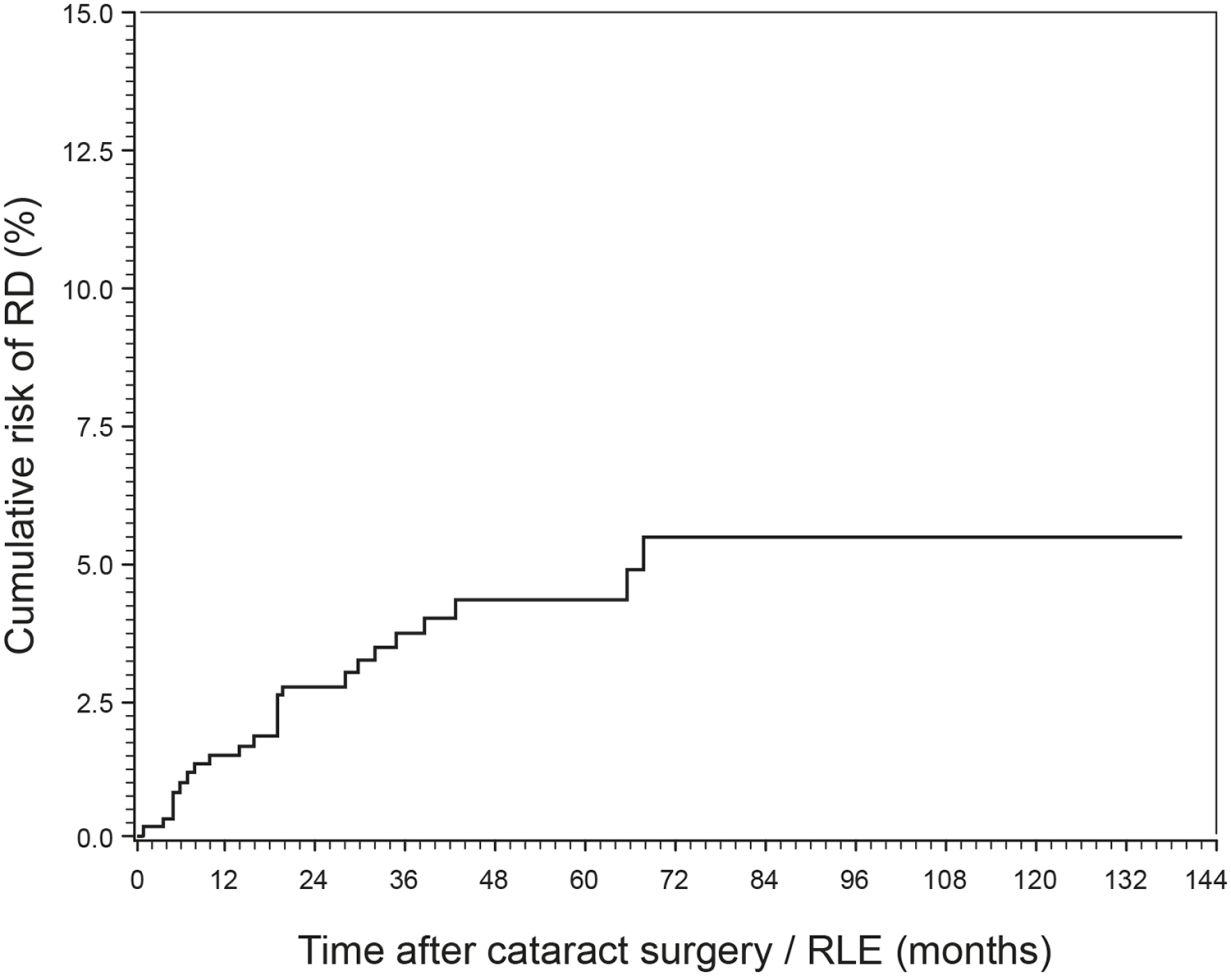
Graph showing the cumulative risk of retinal detachment (RD) during follow-up (full study group). The cumulative risk of RD increased with time up to its maximum after 5.7 years.

From the 677 eyes of the selected age group, 24 cases (22 patients) of RD were identified, resulting in an overall cumulative incidence of 3.55%. Fig 3 shows the cumulative risk of RD during the follow-up period and its increase with time up to a maximum of 5.5% after 5.7 years.

The demographic and clinical characteristics of both groups are compared in Table 1. As compared to the average group of non-RD patients, the group of RD cases was characterized by a longer AL (26.11 vs. 24.29) as the only significant feature. Male gender, age, the number of right and left eyes, and the number of performed cataract extractions vs. RLE showed no significant differences between both groups (Table 1).

**Table 1:** Demographic and clinical characteristics of the non-RD group compared to the RD group.

	non-RD group	RD group	Statistics (*P* value)
**N (eyes)**	653 eyes	24 eyes	
**Males (eyes) (%)**	313 (47.9)	15 (62.5)	n.s. (0.232)
**Females (eyes) (%)**	340 (52.1)	9 (37.5)	
**Age at surgery (years per eye)** Mean ± SD Min–Max	51.1 ± 6.8 13–60	52.0 ± 4.6 40–60	n.s. (0.842)
**Axial length (mm)** Mean ± SD Min–Max	24.29 ± 2.01 20.20–33.97 *	26.11 ± 1.32 23.39–28.86	sign. (< 0.001)
**Cataract surgery, eyes (%)**	565 (86.5)	20 (83.3)	
**RLE, eyes (%)**	88 (13.5)	4 (16.7)	n.s. (0.885)
**Number of RE (%)**	338 (51.8)	14 (58.3)	
**Number of LE (%)**	315 (48.2)	10 (41.7)	n.s. (0.671)

* N = 605 eyes

**Fig 4:**
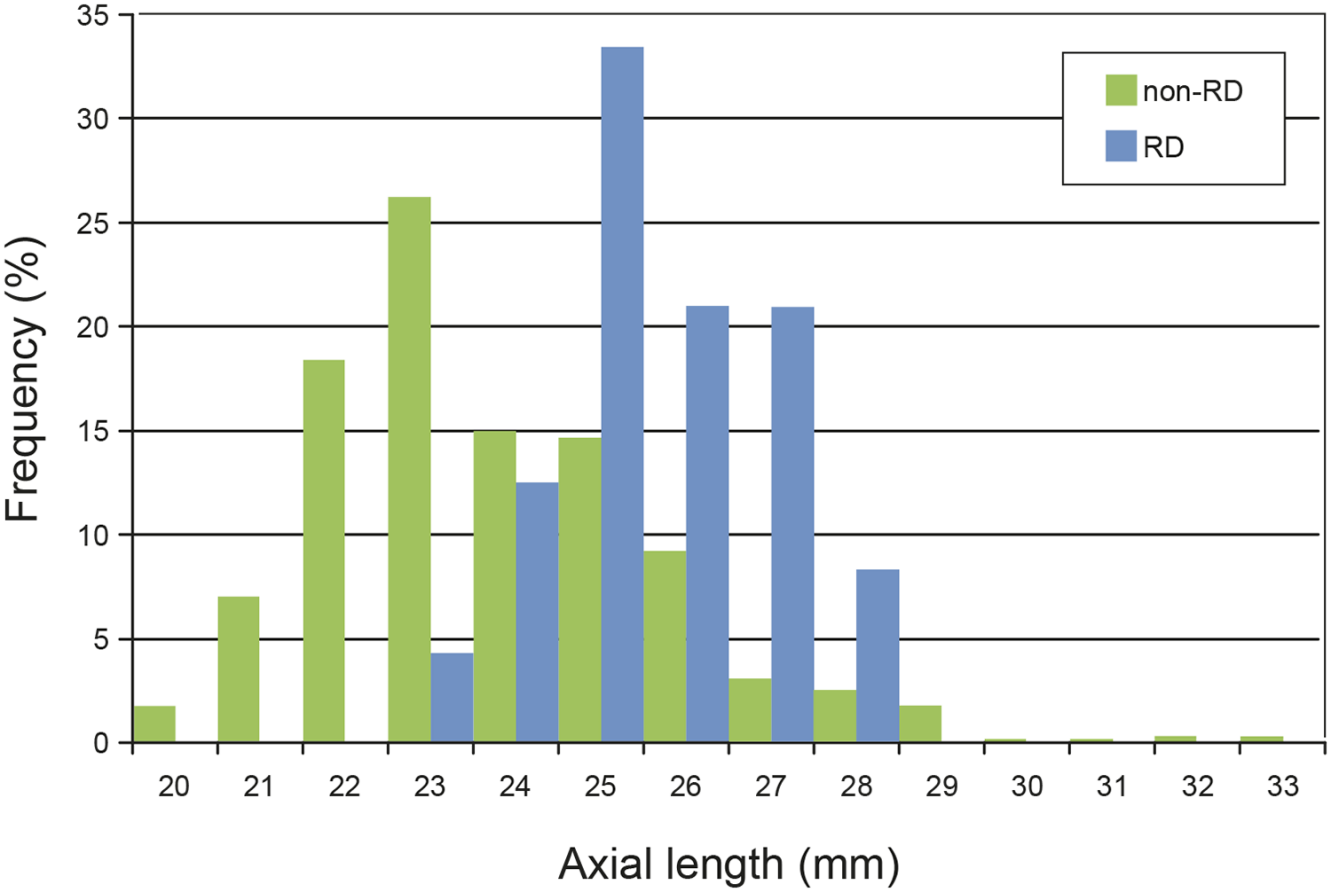
Distribution of axial length in the RD group in comparison to the non-RD group.

**Fig 5:**
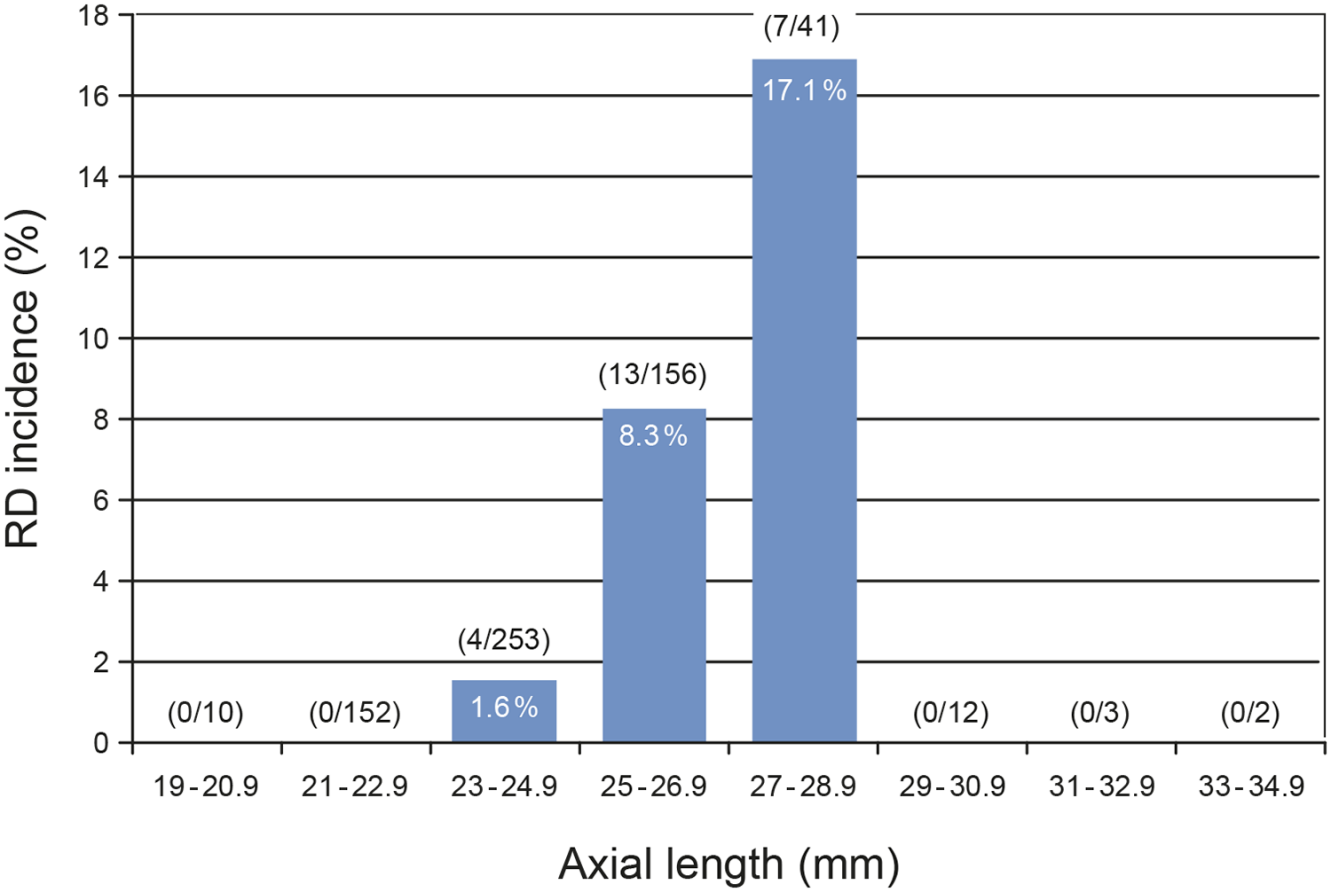
The incidence of RD after cataract surgery or RLE as a function of axial length. The number of RD cases and the number of non-RD eyes per each group of axial length are given in brackets.

**Fig 6:**
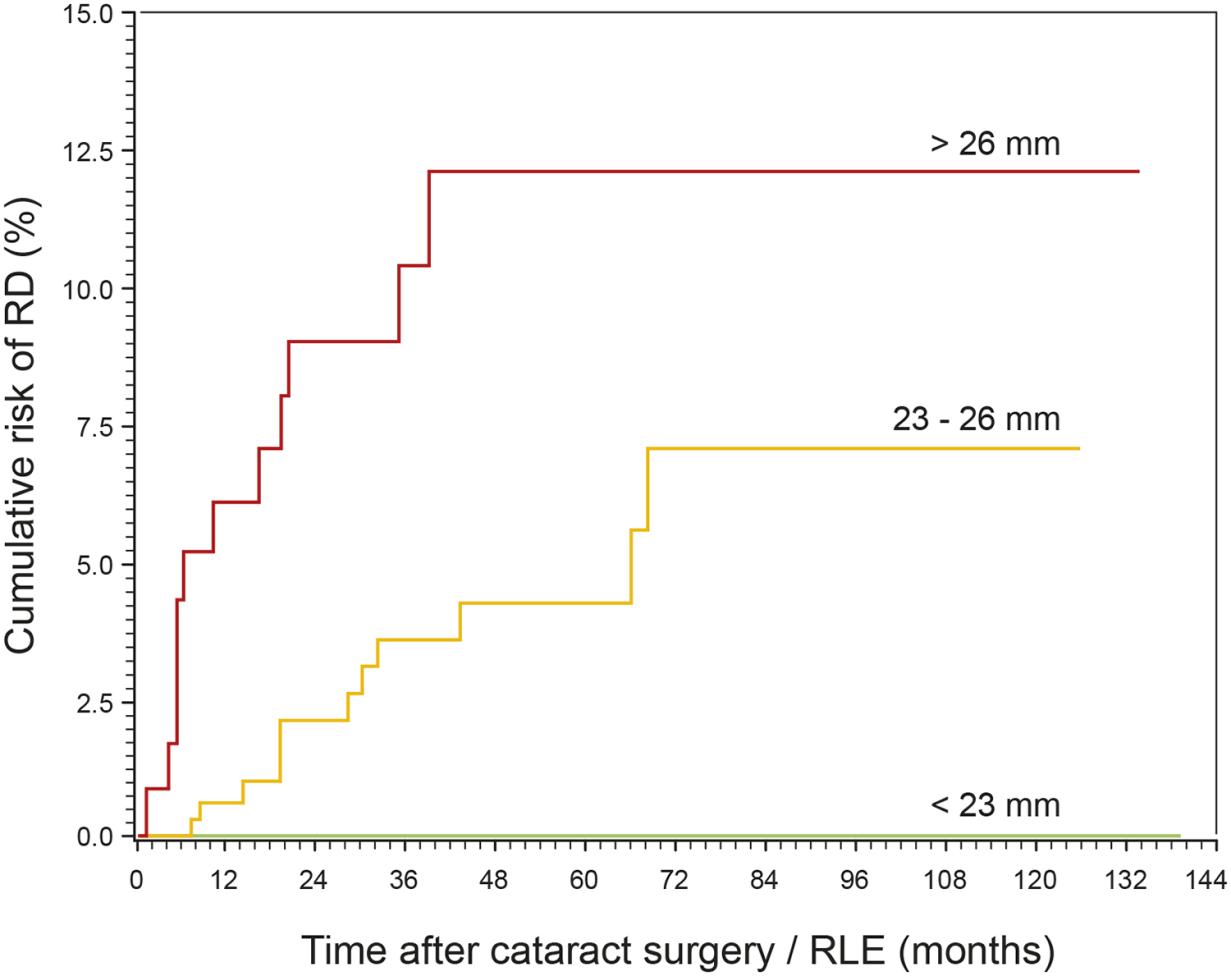
Axial length had a significant effect on the risk of RD after cataract surgery / RLE (P < 0.001). Highly myopic eyes (AL > 26 mm) had the highest risk of postoperative RD (12.25%), followed by eyes of moderate myopia (7.25%). Nonmyopic eyes had no risk of postoperative RD.

Fig 4 shows the distribution of the AL in the RD group compared to the non-RD group. Eighty-three (83) % of the RD cases had an AL of 25 mm or longer. Analysis for AL dependency showed a distinct increase of the RD incidence from its lowest value of 1.6% in eyes of 23 to 24.9 mm length, 8.3% in eyes of 25 to 26.9 mm length, to 17.1% in eyes of 27 to 28.9 mm length (Fig 5). The incidence of RD in the subgroup of 25 to 28.9 mm AL was 10.2%. RD cases did not occur in the 17 eyes with an AL from 29 to 34.9 mm (Fig 5); the mean follow-up of these cases was 3.7 years. Neither the RD group (P = 1.0), nor the non-RD group (P = 0.349) differed significantly in the number of males and females when the groups of moderate myopia (AL 23 to 26 mm) and high myopia (AL > 26 mm) were compared. The mean AL did not differ significantly between males and females in the RD group (mean 26.1 mm in both sexes, P = 0.986), but in the non-RD group (median 24.2 mm in males and 23.6 mm in females, P = < 0.001). AL comparison of eyes in males between the RD and non-RD group showed significant differences (median 26.0 vs. 24.2 mm, P = < 0.001). This was also the case in females (26.2 vs. 23.6 mm, P = 0.001). AL had a significant effect on the risk of RD after cataract surgery or RLE. With an increase of AL by 1 mm the hazard of RD increased by a factor (HR) of 1.42 (P = 0.0001, 95% CI 1.19–1.69). After adjustment for age and sex, the HR per mm AL was 1.44 (P = 0.0001, 95% CI 1.20–1.73). For comparison, the regression without random effect showed a slightly smaller effect of AL of 1.37 (P < 0.0001, 95% CI 1.19–1.59) in the fully adjusted model. Fig 6 shows the cumulative risk of RD after lens extraction during the follow-up period within the subgroups of nonmyopia (AL < 23 mm), moderate myopia (AL 23 to 26 mm) and high myopia (AL > 26 mm). Highly myopic eyes reached a maximum cumulative risk of 12.25% after 3.3 years. The maximum cumulative risk in eyes of moderate myopia was 7.25% after 5.7 years. Nonmyopic eyes with an AL < 23 mm had no risk of postoperative RD.

**Fig 7:**
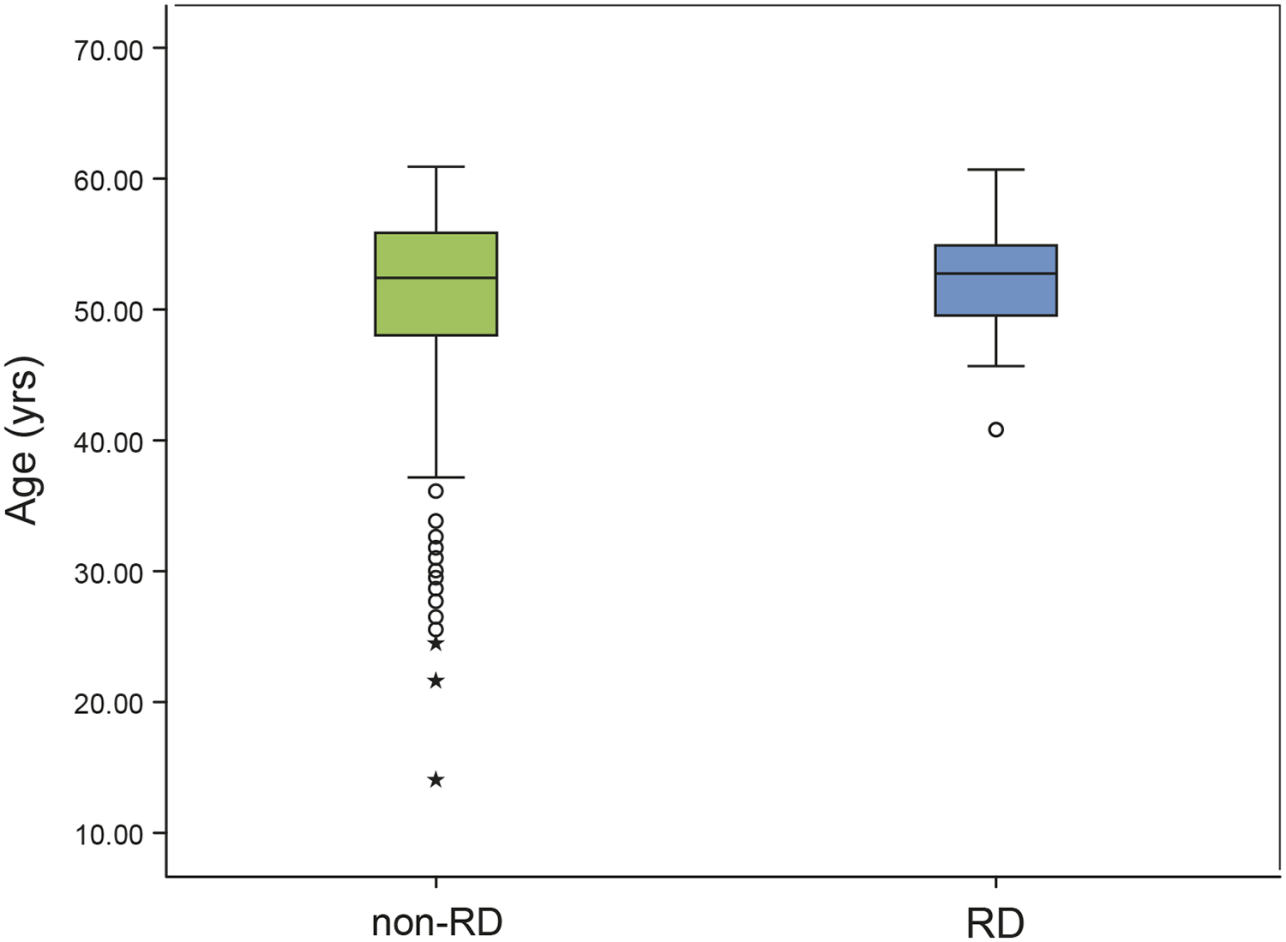
Boxplots of age for the non-RD group and the RD group (each eye counted separately).

**Fig 8:**
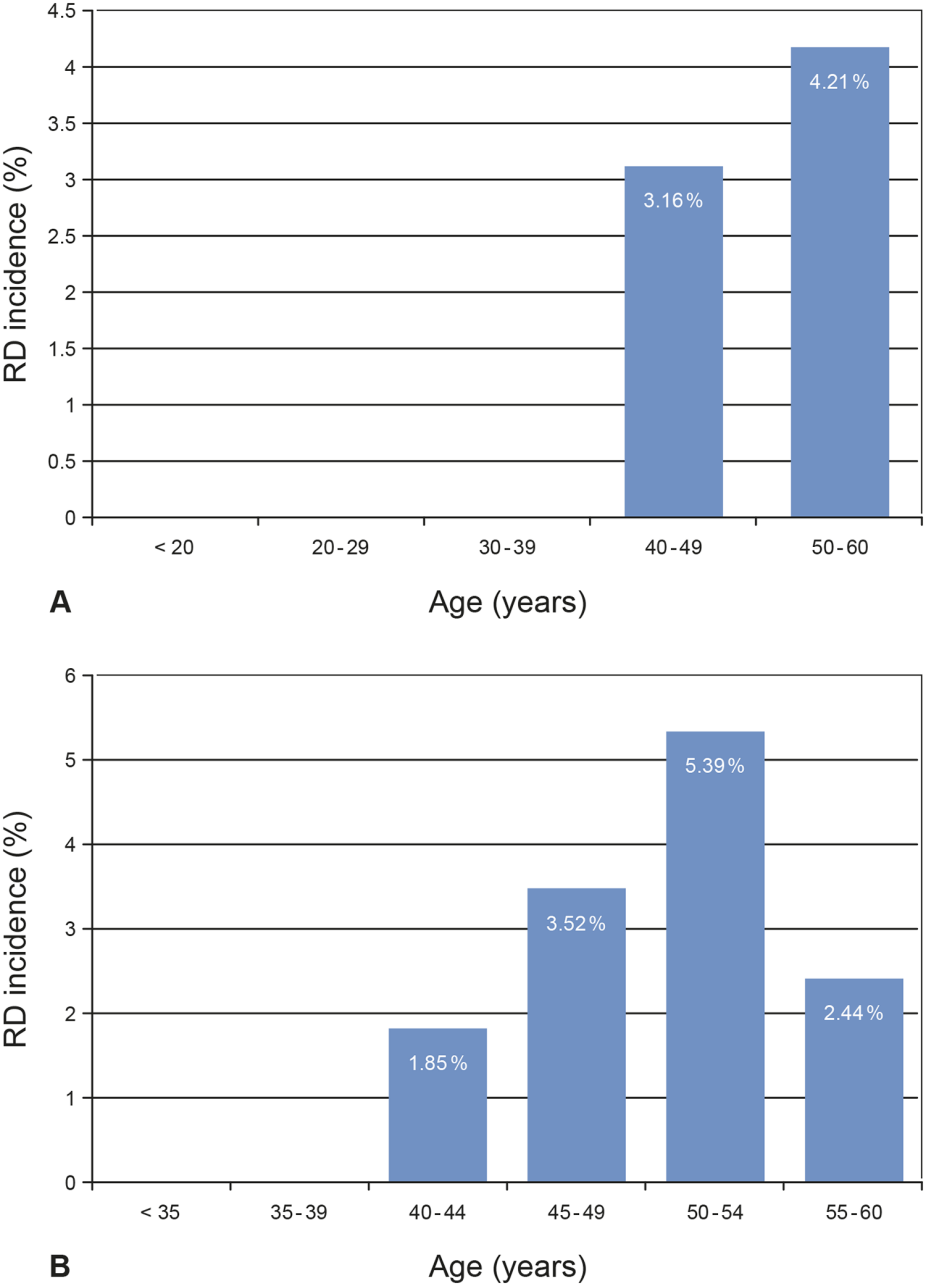
Incidence of retinal detachment (RD) following cataract surgery / RLE as a function of age. (A) Age grouped in decades. (B) Age in half-decade groups.

**Fig 9:**
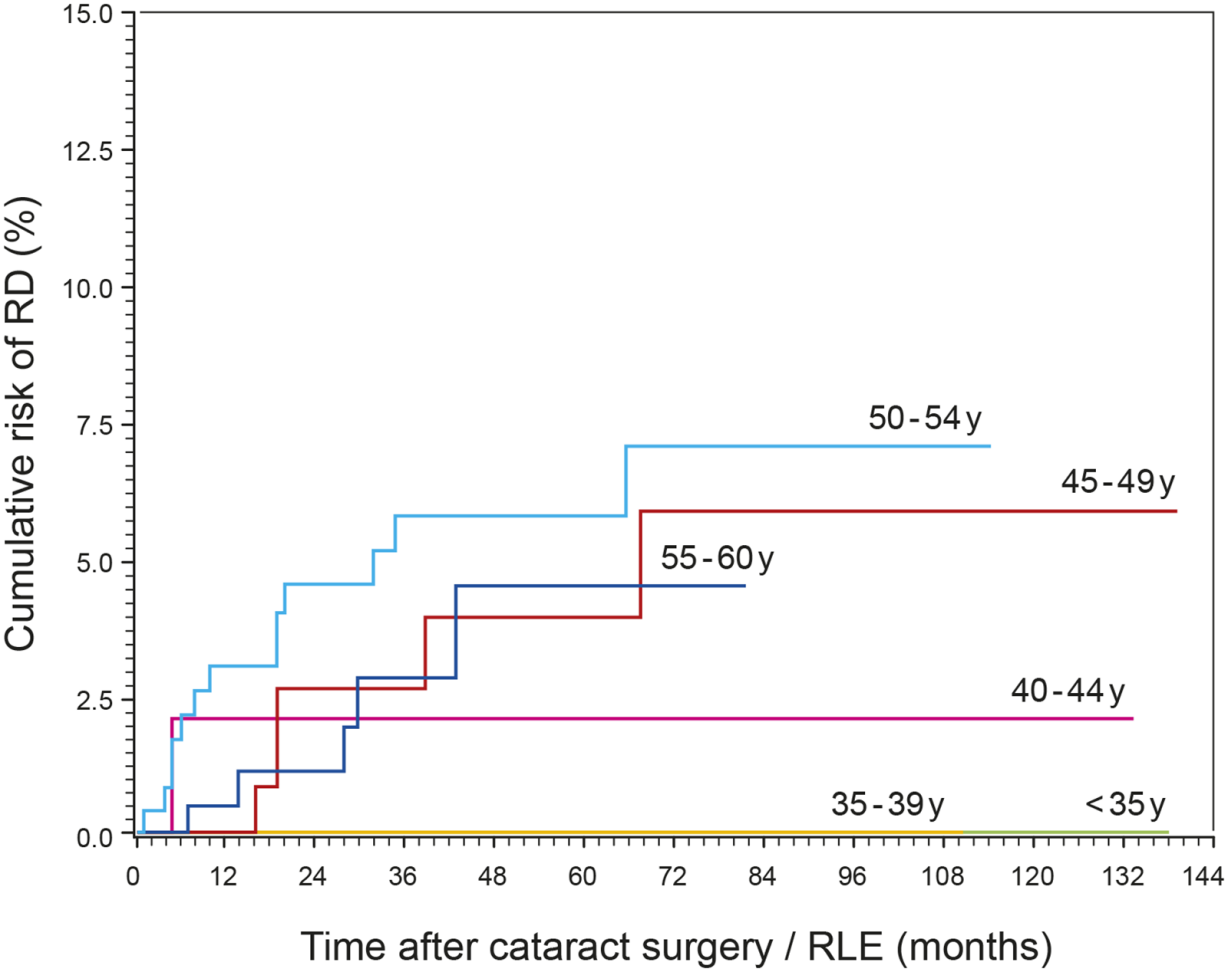
Cumulative risk of RD after cataract surgery / RLE as a function of age. Differences in the risk development were not significant between age categories (P = 0.543).

The boxplots of age for the non-RD group and the RD group are shown in Fig 7. Seventy-five % (75%) of the RD cases were found in the fifth decade and 25% RDs occurred in the fourth decade. Patients younger than 40 years did not develop postoperative RD. Fig 8A shows the incidence of RD as a function of age with 3.16% in the fourth decade and 4.21% in the fifth decade. Further division of the age categories into half decade groups showed that the highest incidence of RD occurred in patients aged 50–54 years (5.39%, Fig 8B), while it was decreasing to 2.44% in patients aged 55–60 years. The second highest RD incidence was found in patients aged 45–49 years (3.52%) and the lowest RD incidence in the age category of 40–44 years (1.85%). Cox-regression analysis showed that with an increase in age of ten years, the hazard to develop RD was not significantly increased by a factor of 1.50 (P = 0.286, 95% CI 0.71–3.15). After adjustment for AL and sex, the HR was 1.51 (P = 0.349, 95% CI 0.64–3.54). Fig 9 illustrates the cumulative risk of the six age categories. Differences in the risk development were not significant between categories (P = 0.543).

**Fig 10:**
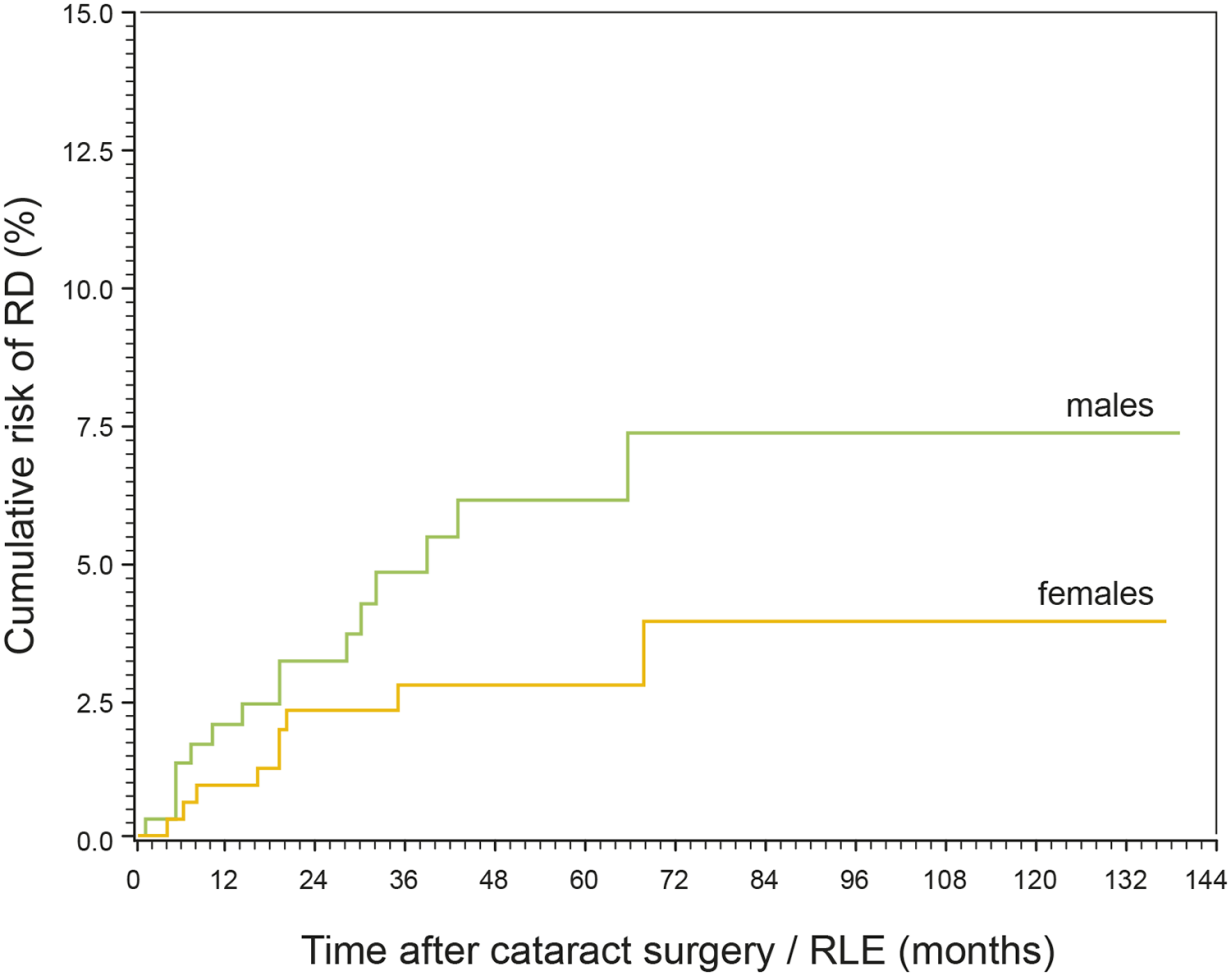
Cumulative risk of RD after cataract surgery / RLE for males and females. The development of RD risk over time is not significantly different between sexes (P = 0.103).

Gender had no significant influence on the RD event rate after phacoemulsification (P = 0.103, Fig 10), though males had an almost twofold risk of developing postoperative RD in comparison to females (HR = 1.96, P = 0.123, 95% CI 0.83–4.63). After adjustment for age and AL, the HR was 1.93 (P = 0.167, 95% CI 0.76–4.88).

Further details on the RD cases are summarized in Table 2. None of the RD cases had a history of RD. One patient had a history of previous RD in the phakic fellow eye. Four cases had a history of retinal degeneration (2 eyes) or preexisting retinal scars (2 eyes) at the area of RD, three of which underwent argon laser coagulation prior to cataract/ RLE surgery. None of the RD cases received preoperative cryocoagulation. Preoperative optical coherence tomography imaging was available for one patient only and showed an attached posterior vitreous body.

**Table 2:** Overview of all cases diagnosed with RD after cataract or clear lens extraction.

					Interval		BCVA (Decimal)					
No (Pt)/Sex/Age at Surg (years)	Status of Macula*/ YAG/History RD/History CC/ ALC	History retinal scars or degen.^1^	AL (mm)	Surgery details^2^, CS or RLE, IOL-type	Surg to RD	Capsul to RD	After CS	After RD	Comment^3^	PPRD	RD surgery	Fellow eye
1 (183)/ M/ 53	+/N/N/N/N	N	27.44	uncompl. phaco., CS, multifocal IOL	5 mo	–	1.00	1.00	RE: rhegm. RD with HT at 10 o’clock	Y	ppV, cercl., gas	pseudophakic
	+/N/N/N/N	N	26.32	uncompl. phaco., RLE, multifocal IOL	10 mo	–	1.00	1.00	LE: rhegm. RD with several tears at 3 o’clock with vitreous traction	Y	ppV, LC, lasercercl., gas	pseudophakic
2 (148)/ M/ 59	+/N/N/N/N	N	25.13	uncompl. phaco., CS, monofocal IOL	14 mo	–	1.00 pp	0.80 pp	RE: RD from 11 to 2 o’clock with tears at 12.30	Y	ppV, PFCL, CC, air, SF_6_	pseudophakic
3 (310)/ M/ 55	+/N/N/N/N	N	25.96	uncompl. phaco., CS, monofocal IOL	28 mo	–	1.00	1.00	RE: RD, large HT at 7 o’clock, small round hole with flap at 8.30 o’clock	Y	ppV, retinotomy, LC, SF_6_	pseudophakic
4 (204)/ M/ 54	-/N/N/N/Y	Y^D^	27.18	uncompl. phaco., CS, monofocal IOL	5 mo	–	0.1	0.05	RE: rhegm. bullous RD, HT at 10.30 o’clock	Y	ppV, cercl., silicone oil	phakic with RD
5 (274)/ M/ 56	+/Y/N/N/N	N	24.54	uncompl. phaco., CS, monofocal IOL	43 mo	0.25 mo	1.0	1.0	RE: low RD	Y	ppV, ILM peeling, gas	pseudophakic
6 (277) M/ 40	+/N/N/N/N	N	27.37	uncompl. phaco., RLE, monofocal IOL	5 mo	–	1.0	1.0	LE: large tear at 11 o’clock and bullous RD over 2 quadrants (I, IV) with beginning PVR	Y	ppV, retinectomy, endodiathermy, LC, SF_6_	pseudophakic
7 (332)/ F/ 54	+/N/N/N/Y	Y^S^	26.42	uncompl. phaco., RLE, multifocal IOL	35 mo	–	1.0	1.0 (6 days after RD surgery)	LE: large HT at 4 o’clock with surrounding RD, PVR reaction and vitreous hemorrhage	Y	ppV, LC, gas	pseudophakic
8 (337)/ F/ 46	+/N/N/N/N	N	26.41	uncompl. phaco., CS, monofocal IOL	16 mo	–	1.0	1.0	LE: HT, RD, vitreous traction	Y	ppV, LC, CC, SF_6_	pseudophakic
9 (382)/ F/ 54	+/N/N/N/N	Y^S^	25.82	uncompl. phaco., RLE, multifocal IOL	19 mo	–	1.0	1.0	RE: HT at 11 o’clock corresponding to existing retinal scar, RD	Y	ppV, SF_6_, LC	pseudophakic
10 (46)/ M/ 48	+/Y/N/N/N	N	27.16	uncompl. phaco., CS, monofocal IOL	39 mo	14 mo	1.0	0.6	RE: temporal HT and RD	Y	ppV, excision of retinal hole, removal of retinal flap, CC, SF_6_	pseudophakic
11 (360)/ F/ 51	-/N/N/N/N	N	28.79	uncompl. phaco., CS, monofocal IOL	20 mo	–	0.6	0.6	LE: vitreous detachment with tear, temporal RD	Y	ppV, endodiathermy, LC, SF_6_	pseudophakic
12 (414)/ M/ 55	+/N/N/N/N	N	23.39	uncompl. phaco., CS, monofocal IOL	30 mo	–	0.8	0.8	RE: RD (2–8 o’clock) with tears at 4 and 6 o’clock reaching the macula; new RD from 6–9 o’clock 14 days after first RD	Y	ppV, LC, CC, SF_6_ re-ppV, LC, SF_6_	pseudophakic
13 (302)/ M/ 53	+/N/N/N/Y	Y^D^	28.86	uncompl. phaco., CS, monofocal IOL	0.7 mo	–	1.0	1.0	RE: superionasal RD with HT at 12 o’clock, vitreous hemorrhage	Y	ppV, LC, CC, SF_6_	phakic
14 (25)/ M/ 50	+/Y/N/N/N	N	25.30	uncompl. phaco., CS, monofocal IOL	66 mo	30 mo	0.9	0.7 (4 days after ppV)	LE: HT at 2 o‘clock, vitreous hemorrhage	Y	LC, ppV for recurrent hemorrhages	pseudophakic
15 (33)/ M/ 60	+/N/N/N/N	N	24.73	uncompl. phaco., CS, monofocal IOL	7 mo	–	0.9	0.4	RE: RD with temporal HT; new RD 16 mo after first RD	Y	cercl., CC, electrolysis	pseudophakic
16 (67)/ F/ 51	+/N/N/N/N	N	25.19	uncompl. phaco., CS, monofocal IOL	8 mo	–	1.0	0.9	RE: rhegm. RD and tear at 12 o‘clock	Y	ppV, lasercercl., SF_6_, LC	pseudophakic
	+/N/N/N/N	N	26.24	uncompl. phaco., CS, monofocal IOL	4 mo	–	0.8	0.8	LE: rhegm. RD, tears at 6 and 9 o‘clock	Y	ppV, cercl., SF_6_, LC	pseudophakic
17 (99)/ M/ 50	-/N/N/N/N	N	27.04	uncompl. phaco., CS, monofocal IOL	19 mo	–	1.0	0.5	RE: RD from 8 to 4 o’clock, HT at 9, 11, and 12 o’clock	Y	ppV, lasercercl., gas	pseudophakic
18 (108)/ F/ 45	+/N/N/N/N	N	24.35	uncompl. phaco., CS, monofocal IOL	19 mo	–	0.9	0.4	LE: giant tear RD and additional holes. Several new RDs and tears after first RD: RD + tears, 5 mo total PVR-RD, 6 mo Re-RD, 8 mo tear, 14 mo	Y	ppV, retinectomy, LC, CC, silicone oil; re-RD surgeries: ppV, LC, SF_6_ ppV, retinectomy, LC, silicone oil ppV-revision under silicone oil LC	phakic
19 (205)/ M/ 47	-/N/N/N/N	N	25.86	uncompl. phaco., CS, monofocal IOL	19 mo	–	1.0	1.0	RE: rhegm. RD from 2 to 8 o’clock, HT at 8 o’clock	Y	ppV, ILM peeling, endodiathermy, SF_6_	phakic
20 (278)/ F/ 54	-/N/N/N/N	N	26.43	uncompl. phaco., CS, monofocal IOL	6 mo	–	0.7	0.05	LE: PVR-RD, PVR-like degeneration at 11 to 12 o’clock, tear at 1 o’clock, massively adherent destroyed vitreous body up to the periphery	Y	ppV, ILM peeling, endodrainage, CC, silicone oil, retinectomy	pseudophakic
21 (84)/ M/ 51	+/N/N/N/N	N	25.30	uncompl. phaco., CS, monofocal IOL	32 mo	–	0.8	0.5 (1 mo after RD surgery)	RE: RD, large HT at 11 o’clock	Y	ppV, LC, gas (air/SF_6_ 20%)	pseudophakic
22 (120)/ F/ 45	+/N/N/N/N	N	25.39	uncompl. phaco., CS, toric IOL	68 mo	–	1.0	CF (directly after RD surgery)	LE: vitreous hemorrhage of unclear origin, small tear at 1 o’clock and surrounding RD	Y	ppV, CC, gas (C_2_F_6_)	phakic

AL = axial length; ALC = argon laser coagulation; BCVA = best corrected visual acuity; Capsul = neodymium:YAG capsulotomy; CC = cryocoagulation; cercl. = cerclage; CF = counting fingers; CS = cataract surgery; FE = fellow eye; HT = horseshoe tear; ILM = inner limiting membrane; IOL = intraocular lens; LC = endolasercoagulation; LE = left eye; mo = months; N = no; No = running number; phaco = phacoemulsification; PPRD = pseudophakic retinal detachment (ie, whether RD considered to be caused by preceding cataract surgery); ppV = pars plana vitrectomy; Pt = patient number according to S1 File; PVR = proliferative vitreoretinopathy; rhegm. RD = rhegmatogenous retinal detachment; RD = retinal detachment; RE = right eye; RLE = refractive lens exchange; Surg = surgery; uncompl. = uncomplicated; Y = yes; YAG = history of posterior neodymium:YAG capsulotomy in eye with RD

*Status of macula at RD (- = macula detached; + = macula attached)

^1^ History of retinal scars ^(S)^ or retinal degeneration ^(D)^ in the area of later retinal detachment

^2^ Surgical complications or comments

^3^ Information available on retinal detachment (preoperative or intraoperative) and postoperative course

All RD cases had uncomplicated phacoemulsification and implantation of an intraocular lens. Nineteen of the implanted IOLs were monofocal, four were multifocal, and one IOL was toric. The fellow eye was pseudophakic in 19 cases and phakic in 5 cases. Neodymium:YAG laser capsulotomy was performed in three eyes with a time interval previous to RD of 0.25, 14, and 30 months, respectively.

**Fig 11:**
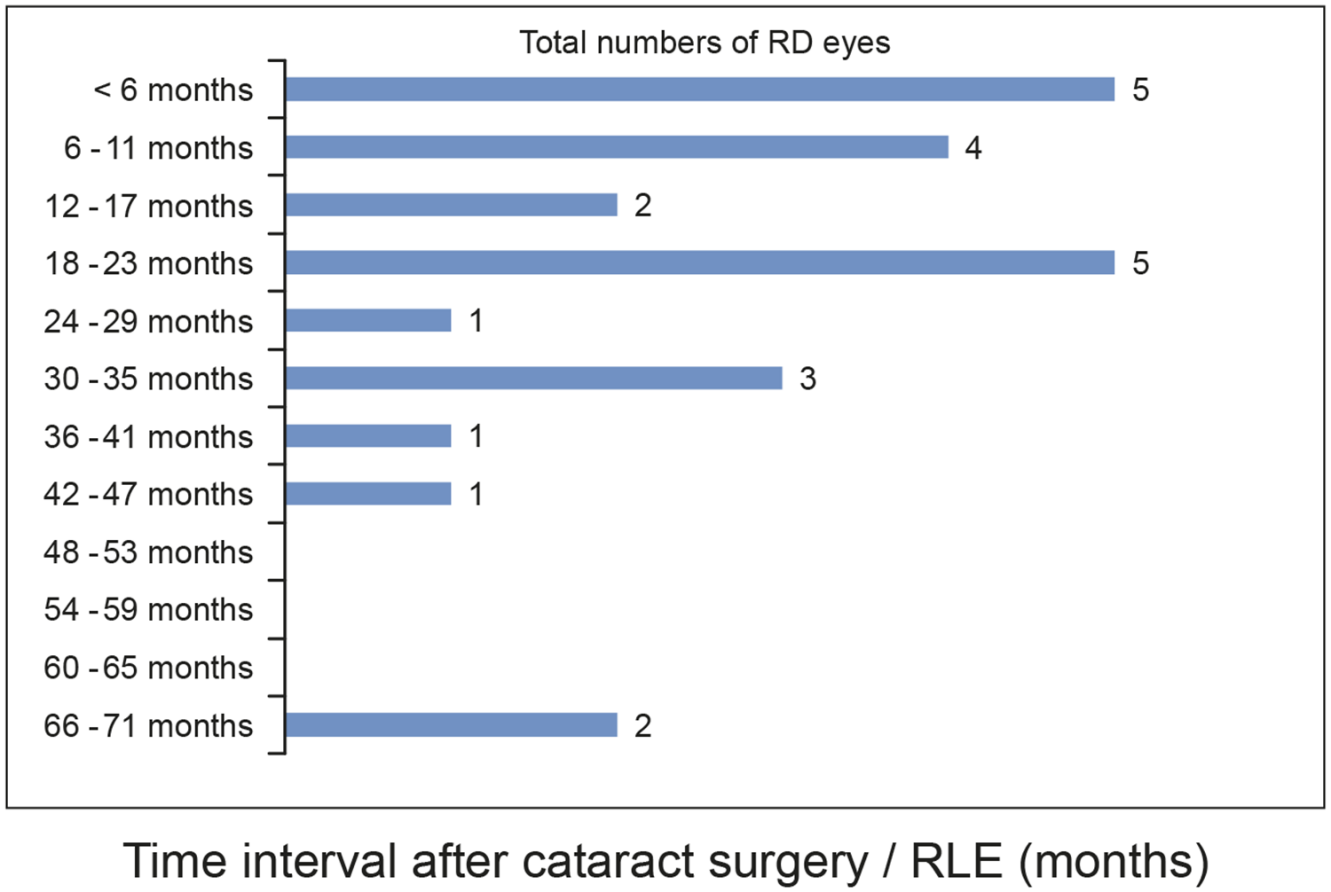
Bar graph of the total number of RD cases according to the postoperative time interval.

The time interval between cataract surgery or RLE and RD varied from 0.7 to 68 months (mean 21.57, SD ± 18.36; Table 2, Fig 11). Twenty-five (25) % of all RDs occurred within 6 months after cataract extraction or RLE, 38% during the first postoperative year, and 67% during the first 2 years; 92% of all RDs occurred within 3.6 years from surgery, and two cases developed RD 5.5 and 5.7 years after surgery (Fig 11). The two cases of late RD were not associated with higher AL (Table 2).

Two patients (1 male, 1 female) developed bilateral RD and 20 patients showed unilateral RD with 12 cases of RD in the right eye (11 males, 1 female), and 8 RD cases in the left eye (2 males, 6 females).

Horseshoe tears, tears, retinal holes, and RD occurred predominantly in the superior hemisphere (13 eyes), in contrast to 5 eyes with an inferior retinal detachment. The macula was mostly attached at the time of RD (19 eyes), but detached in 5 eyes (Table 2).

The treatment of RDs included pars plana vitrectomy (PPV), retinectomy, retinotomy or inner limiting membrane peeling as required, and pneumatic retinopexy combined with cryo- and/ or laserretinopexy in most of the cases. Silicone oil tamponade was applied in 3 eyes. PPV with additional cerclage was applied in 3 eyes, and a scleral buckle surgery instead of PPV has been performed in 1 eye (Table 2).

The mean BCVA after RD surgery followed by a minimum recovery time of three months was 0.26 logMAR (SD ± 0.39) and did not differ significantly from the corresponding values after cataract/ RLE surgery (P = 0.109; Table 2). In 54.2% of the cases the BCVA obtained after cataract/ RLE surgery was achieved again following the RD treatment.

## Discussion

This study confirmed that the cumulative incidence of RD after cataract surgery is higher than the incidence in the general population that varies between 0.005% and 0.0179% [15–19]. The overall cumulative incidence of RD of 3.55% observed in our study was in the upper range from 0.0% to 3.6% of RD incidences reported by other studies using phacoemulsification for cataract extraction [4, 22–28]. However, the incidence of RD from our results is not comparable to the literature data as we focused on a young-aged patient group of below 61 years. As young age is a known risk factor for RD following cataract surgery [1, 4, 6–8, 28], a higher incidence of RD was to be expected.

We found the highest incidence of postoperative RD in the patients age of the fifth decade (4.21%), followed by the fourth decade (3.16%; Fig 8A). This corresponds to findings of a retrospective study of 12,222 consecutive cases of cataract surgery where the highest incidence of RD was observed in patients of the fifth decade (2.1%), followed by those of the fourth decade (around 1.50%) [28]. In a nationwide study from Taiwan, as well as in a study on a Danish patient sample, the highest incidence of RD was also found in patients of the fifth decade [29, 30]. According to other studies, highest RD incidences were found in patients younger than 50 years, followed by those of the fifth decade [1, 2, 10]. While in our study none of the patients younger than 40 years developed postoperative RD, other studies reported the occurrence of RD in this age group with generally lower incidences than in patients older than 40 years [3, 7, 28, 29]. We found that division of the age groups to half decade groups showed a distinct decrease of incidence in patients aged 55–60 years (2.44%) and a peak of RD cases in the 50–54 years group (5.39%; Fig 8B). According to a number of studies on pseudophakic RD, patients older than 60 years showed generally the lowest incidences of RD as compared to the younger age groups [1, 2, 8, 10, 28, 31]. Young age might be a risk factor for pseudophakic RD as posterior vitreous detachment (PVD) has not yet been completed; whereas in older patients the completed process of PVD is a protective factor against RD [7, 19, 32].

Moreover, cataract surgery itself is a risk factor for RD. Lens exchange to a distinctively thinner artificial lens enlarges the volume of the vitreous cavity and the vitreous body is shifted anteriorly [32]. Additionally, the removal of the natural lens disturbs the barrier function of the lens between the anterior and posterior segment and induces biochemical changes of the vitreous body [33]. These modifications seem to expedite destabilization of the vitreous body and to promote PVD that may be related to the onset of postoperative RD [20, 34].

High myopia is a major risk factor for the occurrence of pseudophakic RD [1–3, 11, 28]. In our study, axial length was the only feature showing significant differences between the RD and the non-RD group (Table 1). The distribution of axial lengths in the RD group was comparable to the findings of Olsen and Jeppesen (2012) [28] with highest numbers of RD cases in eyes of 25 to 27 mm of AL (Fig 4). Also, similarly to the study of Olsen and Jeppesen (2012) [28] the highest incidence of postoperative RD was found in eyes from 27–28.9 mm of AL (Fig 5). It was remarkable that in the 17 eyes longer than 29 mm no RD occurred. The mean follow-up of these eyes was 3.7 years and thus in the range where the majority of RD cases occurred. Other studies documented pseudophakic RD in this AL spectrum [7, 28].

We found that with an increasing AL the cumulative risk of RD after surgery was also increasing, which is in accordance to other studies as well [1, 2]. We observed the highest cumulative risk in eyes with an AL of ≥ 26 mm after 3.3 years (Fig 6) which corresponds to the results of Sheu et al. (2006) [1]. Another study of Sheu et al. (2010) [2] described a distinct late increased risk of RD in this AL group after four years reaching a maximum after eight years. We have not noticed such a late increased risk, though the follow-up time in our study was even longer (11.7 years). The maximum cumulative risk of 12.25% in this AL group is higher than that found by Sheu et al. (2006 (2.5%) [1] and 2010 (6.5%) [2]), which can be attributed to the selected group of young-aged patients in our study, that have already a higher risk of postoperative RD.

According to the results of the present study the eyes of male patients had an almost twofold risk of developing postoperative RD in comparison to females (HR = 1.96, P = 0.123, 95% CI 0.83–4.63) though gender did not significantly influence the pseudophakic RD event rate (Fig 10). This is in accordance to other studies where male sex has frequently been reported as a risk factor for RD after cataract surgery [8–10, 29, 35, 36]. Anatomic differences of the eye and vitreous have been discussed as potentially contributing factors for a higher risk of pseudophakic RD in males [8]. Men have longer axial lengths [37–39] and tend to undergo later PVD in comparison to women [40]. An earlier PVD in women may protect from RD after cataract surgery. It has also been discussed that men are under higher risk of pseudophakic RD as they may be more likely to experience eye trauma due to occupational or lifestyle activities [8, 28, 32].

In our study, 92% of all RDs occurred within 3.6 years from cataract or RL extraction and the longest time interval was 5.7 years after surgery. While some authors questioned the relation of RD to cataract surgery for cases occurring later than 4 years postoperatively [7, 41], and have not found a significant late increase of pseudophakic RD [5], a significant late increased risk of RD was found especially in male patients with high myopia in a study from Taiwan [2]. These authors described an increase of risk for RD from 0.5% after 4 years to 6.5% after 8 years for this patient group [2]. We consider all of the observed RD cases in our study to be related to the preceding lens extraction surgery. The two cases of late RD (5.5 and 5.7 years postoperative, Table 2) were one male and one female patient of moderate myopia (below 26 mm) but both of the two age groups with highest pseudophakic RD risk.

Treatment of pseudophakic RD has considerably changed in the past years and major improvements were made in modern vitreoretinal surgical techniques [32]. PPV is superior to scleral buckling regarding the anatomical outcome (retinal reattachment) and redetachment rates, but differs not with respect to BCVA improvement [36]. According to the VIPER study, vitrectomy with gas is an efficient and safe treatment for pseudophakic RD and an additional encircling band does not significantly reduce the risk for retinal redetachment [42]. In our study, PPV was performed in 23 of the RD cases and scleral buckle surgery was performed in one case in which a redetachment occurred 16 months after the treatment (Table 2). Moreover, the BCVA decreased in the latter case from 0.9 after cataract surgery to 0.4 after RD treatment. The mean final BCVA of all RD cases was 0.26 logMAR in our study, which was distinctively better than the corresponding value of 0.42 logMAR in the SPR study [36]. The functional outcomes of other studies vary between final BCVA of 0.28 [43] and 0.61 logMAR [44].

Possible limitations of our study are the retrospective design and the lack of a control group. In addition, the inclusion of a higher number of patients could have been achieved by a multicenter approach of the study. However, the single-center design of our study has the advantage that all surgeries were performed by the same surgeon. As postoperative RD occurred also after 68 months, long follow-up periods for the whole study population should be attempted in future studies.

Our findings showed that patients with axial lengths between 25 and 29 mm have a significant risk of pseudophakic RD and require careful consideration of the need for cataract surgery or RLE. If patients in our study with these axial lengths would not have undergone lens surgery, 83.3% of all RD cases of our study could have been avoided with only 29.1% fewer lens extractions performed. Moreover, lens extraction in patients aged 50–54 years, of male gender, and with preexisting retinal findings should be avoided or delayed. We recommend careful screening for retinal findings prior to cataract extraction and a long-term close follow-up of the fundus in patients with high postoperative RD risk where surgery cannot be delayed.

## Supporting information

S1 File Supplementary excel file with raw data. (XLSX)
